# Lateral distribution of electrical defect concentration in SA TG coplanar IGZO TFTs extracted via low frequency noise analysis

**DOI:** 10.1038/s41598-026-58574-z

**Published:** 2026-06-21

**Authors:** Dong-Ho Lee, Jin-Ha Hwang, Chae-Eun Oh, Jihee Ryu, Jae-Man Jang, Byung-Du Ahn, Jong-Uk Bae, Hyuck-In Kwon

**Affiliations:** 1 Major in Intelligent Semiconductor Engineering, Chung-Ang University, 84 Heukseok-ro, Dongjak-gu, Seoul, Korea; 2 Research and Development Center, LG Display Company, Paju, 10845 Korea

**Keywords:** SA TG coplanar IGZO TFTs, Low frequency noise, Lateral distribution, Gate insulator trap density, Effective channel length, Engineering, Materials science, Nanoscience and technology, Physics

## Abstract

This study presents a methodology for extracting the lateral distribution of electrical defect concentration (*N*_B_) associated with gate-insulator traps contributing to low-frequency noise (LFN) of self-aligned top-gate (SA TG) coplanar indium–gallium–zinc oxide (IGZO) thin-film transistors (TFTs) using low-frequency noise (LFN) analysis. The proposed approach utilizes modulation of the effective channel length by drain voltage (or source voltage) together with an LFN power spectral density model to obtain the average defect concentration corresponding to different effective channel lengths, from which the lateral defect distribution can be derived. The extracted results reveal that pristine devices exhibit a relatively uniform defect distribution along the channel with slightly higher values near the source and drain regions. After the application of saturation stress, a noticeable increase in the defect concentration is observed near the drain side of the channel, indicating localized degradation of the gate insulator. These findings demonstrate that LFN analysis can be used to experimentally probe the spatial distribution of gate-insulator defects in SA TG coplanar IGZO TFTs, providing a useful diagnostic approach for studying reliability degradation mechanisms in advanced display backplane devices.

**Supplementary Information:**

The online version contains supplementary material available at https://doi.org/10.1038/s41598-026-58574-z.

## Introduction

Oxide semiconductor thin-film transistors (TFTs) have become an essential component of modern display backplanes due to their suitability for large-area fabrication and stable operation under low-temperature processing conditions^1–4^. Among them, indium–gallium–zinc oxide (IGZO) TFTs are extensively utilized in active-matrix organic light-emitting diode (AMOLED) displays, where stringent requirements on electrical stability and long-term reliability must be satisfied^5,6^. Because the electrical performance of backplane TFTs directly affects display uniformity and operational lifetime, understanding the factors that govern device reliability is of critical importance. In advanced OLED backplane technologies, self-aligned top-gate (SA TG) coplanar IGZO TFTs are frequently employed to mitigate parasitic effects associated with gate-to-source/drain overlap and to improve device scalability^7–10^. However, this device architecture inherently requires a self-aligned gate-insulator etching process to define the source/drain access regions^11–13^. During this fabrication step, the gate insulator outside the gate electrode is directly exposed to plasma, whereas the gate insulator beneath the gate remains shielded. As a result, plasma-induced charging or etch damage has been reported to generate oxide charges, slow oxide traps, and near-interface traps in SiO_2_-based gate dielectrics in MOS structures^14,15^. Although the device structure differs from the present IGZO TFTs, these reports support the possibility that plasma-exposed gate-insulator regions can contain electrically active oxide defects. In OLED applications, IGZO TFTs are often operated under conditions that impose relatively high lateral electric fields near the drain region. Such operating conditions can induce additional localized degradation of the gate insulator, superimposed on the fabrication-induced non-uniformity. Consequently, the electrical quality of the gate insulator is expected to vary laterally along the channel, rather than remaining spatially uniform across the device. Since gate-insulator-related defects play a decisive role in determining the electrical characteristics and reliability of IGZO TFTs^16,17^, an accurate evaluation of not only their magnitude but also their spatial distribution is essential. Previous LFN studies have shown that noise characteristics are sensitive to trap-related degradation and geometry-dependent defect effects in TFTs, including oxide TFTs under bias stress, poly-Si TFTs under self-heating stress, and elevated-metal metal-oxide TFTs^18–20^. These results support the use of LFN as an electrical probe for spatially non-uniform trap-related degradation. In particular, locally degraded regions of the gate insulator can dominate device behavior, making channel-averaged assessments of gate-insulator quality insufficient. Nevertheless, to the best of our knowledge, no prior studies have explicitly reported the quantitative extraction of the lateral distribution of electrical defect concentration in the gate insulator of IGZO TFTs. While charge-pumping-based techniques have been widely used in silicon Metal–Oxide–Semiconductor Field-Effect Transistors (MOSFETs) to investigate the spatial distribution of gate insulator defects and stress-induced degradation^21–25^, such approaches are difficult to apply directly to IGZO TFTs because channel inversion and the recombination mechanisms assumed in conventional charge-pumping methods are not well defined in accumulation-mode oxide TFTs. In this work, low-frequency noise (LFN) analysis is employed to extract the lateral distribution of electrical defect concentration in the gate insulator of SA TG coplanar IGZO TFTs, and the extracted distributions are systematically compared before and after saturation stress.

## Method

**Figure 1:**
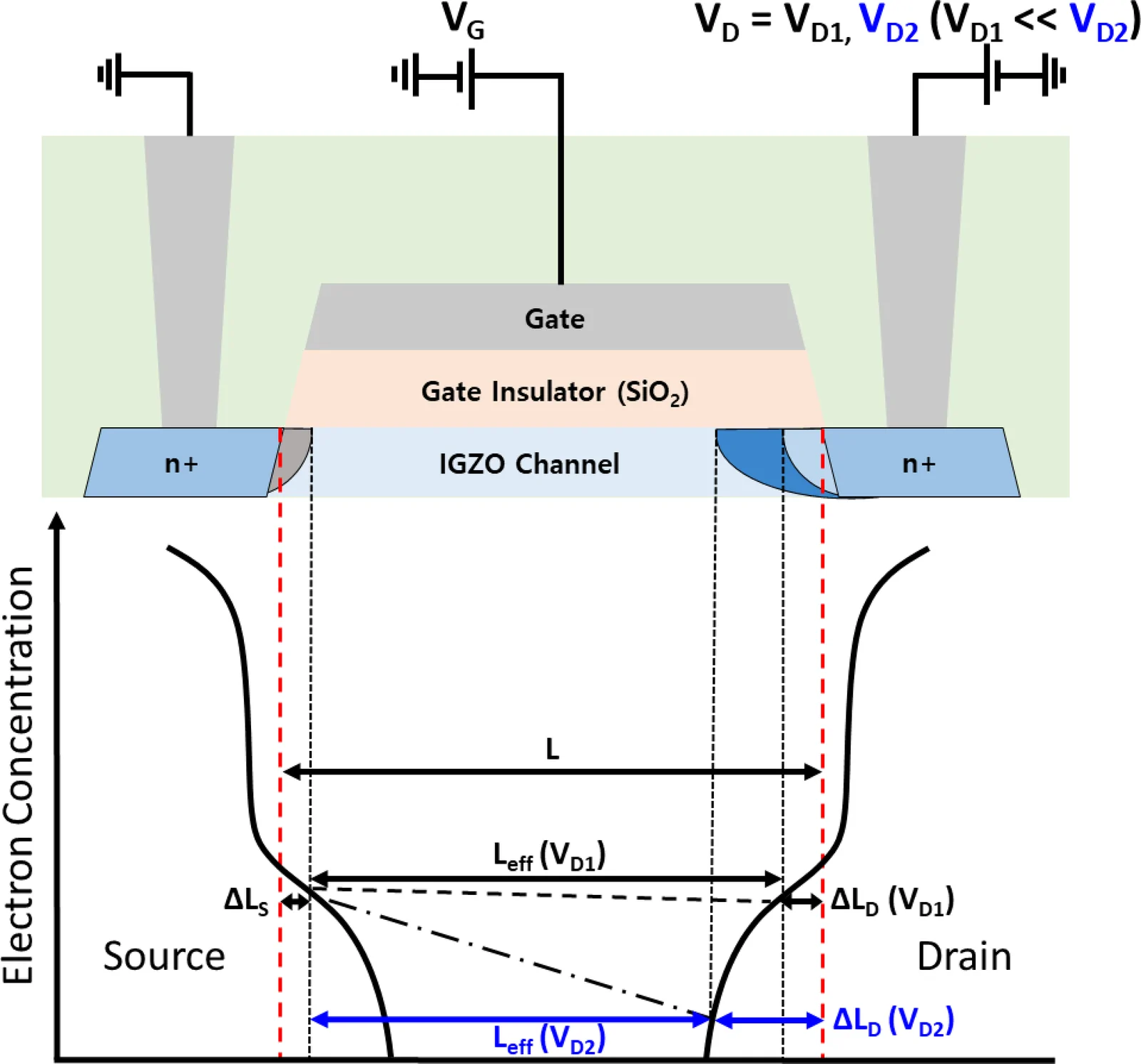
Cross-sectional view of the SA TG coplanar IGZO TFT and the schematic of electron concentration profile along the channel. The solid line indicates the equilibrium electron concentration, and the dotted lines represent the gate-induced electron concentration modulated by *V*_D1_ and *V*_D2_ (*V*_D2_ > *V*_D1_). *ΔL*_S_ and *ΔL*_D_ denote the source- and drain-side channel shortening, respectively.

In the proposed method for extracting the spatial distribution of the electrical defect concentration in the gate insulator of SA TG coplanar IGZO TFTs, the channel shortening (*ΔL*) and the parasitic resistance (*R*_par_) are first extracted by varying the drain voltage (*V*_D_), or equivalently the source voltage (*V*_S_), under a fixed gate voltage (*V*_G_) while the device is operated in the linear regime. Figure 1 presents a cross-sectional view of the SA TG coplanar IGZO TFT and a schematic of the electron concentration profile within the channel. In this architecture, the source/drain extension regions are n^+^-doped to minimize *R*_par_. During the subsequent annealing stage, high-density free electrons from these extensions migrate into the IGZO layer, resulting in an elevated electron concentration in the channel regions adjacent to the source and drain^26–29^. In our previous report, *ΔL* and *R*_par_ were extracted as functions of the applied *V*_G_ at low *V*_D_ levels^30^. Similarly, by varying the magnitude of *V*_D_ while *V*_G_ remains fixed, the drain-side channel shortening (*ΔL*_D_) and the corresponding parasitic resistance (*R*_par,D_) can be modulated. This is because an increase in *V*_D_ at a constant *V*_G_ reduces the gate-to-drain voltage (*V*_GD_ = *V*_G_ − *V*_D_), thereby decreasing the *V*_GD_-induced electron concentration near the drain electrode. In Fig. 1, the solid lines denote the equilibrium electron concentration (*n*_0_), while the two dotted lines represent the *V*_GD_-induced electron concentration at *V*_S_ = 0 V under two drain biases, *V*_D1_ and *V*_D2_ (*V*_D2_ > *V*_D1_). The effective channel boundaries are defined by the positions where the gate-induced electron concentration equals *n*_0_. *ΔL*_S_ represents the source-side channel shortening. Because the source-side potential remains unchanged when *V*_D_ is varied (with *V*_S_ = 0 V), *ΔL*_S_ and the corresponding parasitic resistance *R*_par,S_ are essentially independent of *V*_D_. In contrast, *ΔL*_D_ increases as *V*_D_ increases due to the reduction in *V*_GD_, making both *ΔL*_D_ and the associated drain-side parasitic resistance *R*_par,D_ functions of *V*_D_. The total effective channel length (*L*_eff_) and *R*_par_ are therefore expressed as

1 $$L_{eff} \left( {V_{D} } \right) = L - \left( {\Delta L_{S} + \Delta L_{D} \left( {V_{D} } \right)} \right)$$

and

2 $$R_{par} \left( {V_{D} } \right) = R_{par,S} + R_{par,D} \left( {V_{D} } \right)$$

where *L* is the drawn gate length. By symmetry, when *V*_D_ = 0 V and *V*_S_ is varied, modulation occurs on the source side through changes in the gate-to-source voltage *V*_GS_, affecting *ΔL*_S_ and *R*_par,S_ in an analogous manner, while the drain-side parameters remain nearly constant. To extract the magnitude of *ΔL*_S_, *ΔL*_D_(*V*_D_), *R*_par,S_, and *R*_par,D_(*V*_D_) at a fixed *V*_G_, the paired *V*_D_-based transmission line method (TLM)^31,32^ can be employed. In the SA TG coplanar TFTs, the total resistance between the source and drain electrodes measured in the linear region at a fixed *V*_G_ (*R*_T_) can be expressed using the following equation:

3 $$R_{T} = \frac{{V_{D} }}{{I_{D} }} = R_{par,S} + R_{par,D} \left( {V_{D} } \right) + \frac{{L - \left( {\Delta L_{S} + \Delta L_{D} \left( {V_{D} } \right)} \right)}}{{\mu_{FEi} C_{i} W\left( {V_{G} - V_{TH} - \raise.5ex\hbox{$\scriptstyle 1$}\kern-.1em/ \kern-.15em\lower.25ex\hbox{$\scriptstyle 2$} V_{D} } \right)}}$$

where *I*_D_ is the drain current, *μ*_FEi_ is the intrinsic field-effect mobility, *C*_i_ is the gate insulator capacitance per unit area, *W* is the channel width, and *V*_TH_ is the threshold voltage, respectively. In the paired *V*_D_-based TLM, the quantities (*ΔL*_S_ + *ΔL*_D_(*V*_D_)) and (*R*_par,S_ + *R*_par,D_(*V*_D_)) at a given *V*_G_ are extracted from the intersection of two straight lines obtained at two closely spaced drain voltages, *V*_D_ ± *ΔV*_D_, where *ΔV*_D_ is sufficiently small to maintain operation in the linear regime. At *V*_D_ = 0 V, symmetry requires that *ΔL*_S_ = *ΔL*_D_ and *R*_par,S_ = *R*_par,D_. Using this condition as a reference, the individual components *ΔL*_S_, *ΔL*_D_(*V*_D_), *R*_par,S_, and *R*_par,D_(*V*_D_) can be separated and determined as functions of *V*_D_ under a fixed *V*_G_. To verify whether the parasitic resistance of the same device changes after saturation stress, a modified external loading method (MELM) was additionally employed as a single-device extraction method^33^. The MELM-extracted *R*_par_ was first compared with the *V*_G_-based TLM result for devices fabricated under the same process conditions, and the two methods showed good agreement, as summarized in Fig. S1(b) (Supplementary Information).

**Figure 2:**
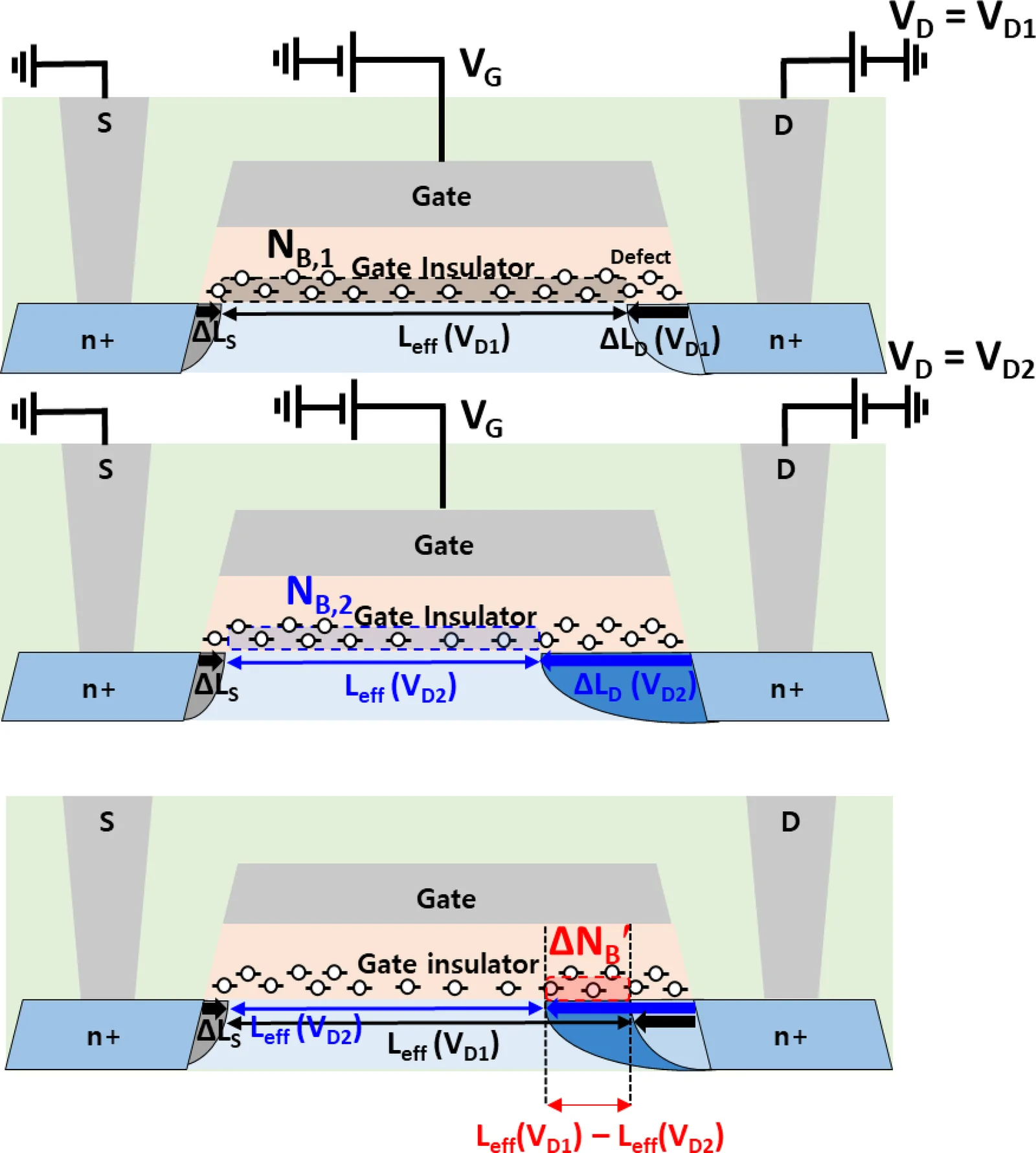
Conceptual diagram of the proposed methodology for extracting the lateral distribution of *N*_B_ in the gate insulator. By varying *V*_D_, the *L*_eff_ is modulated (*L*_eff_(*V*_D2_) < *L*_eff_(*V*_D1_)). The average defect concentration within a specific segment is obtained by comparing the extracted *N*_B_ values at different *L*_eff_.

Figure 2 illustrates the conceptual diagram of the proposed method for extracting the spatial distribution of the electrical defect concentration in the gate insulator of SA TG coplanar IGZO TFTs. As discussed in Fig. 1, when *V*_G_ is fixed, the magnitude of *V*_D_ modulates the drain-side channel shortening, thereby changing the effective channel length *L*_eff_. Consequently, different drain biases (*V*_D2_ > *V*_D1_) yield different effective channel lengths (*L*_eff_(*V*_D2_) < *L*_eff_(*V*_D1_)). If the average defect concentration in the gate insulator above the effective channel region, denoted as *N*_B_, can be extracted for a given *L*_eff_, then the spatial distribution of *N*_B_ can be reconstructed. Specifically, under two drain bias conditions, the extracted values *N*_B1_ and *N*_B2_ correspond to the average defect concentrations over the effective channel lengths *L*_eff_(*V*_D1_) and *L*_eff_(*V*_D2_), respectively. From the definition of an average value,

4 $$N_{B1} = \frac{1}{{L_{eff} \left( {V_{D1} } \right)}}\mathop \smallint \limits_{0}^{{L_{eff} \left( {V_{D1} } \right)}} N_{B} \left( x \right)\,dx,\quad N_{B2} = \frac{1}{{L_{eff} \left( {V_{D2} } \right)}}\mathop \smallint \limits_{0}^{{L_{eff} \left( {V_{D2} } \right)}} N_{B} \left( x \right)\,dx$$

The average defect concentration within the segment *L*_eff_(*V*_D2_) < *X* < *L*_eff_(*V*_D1_), denoted as *N′*_*B*_(*L*_eff_(*V*_D2_), *L*_eff_(*V*_D1_)) can therefore be obtained as

5 $$N_{B}^{\prime } (L_{eff} \left( {V_{D2} } \right), L_{eff} \left( {V_{D1} } \right) = \frac{{N_{B1} \cdot L_{eff} \left( {V_{D1} } \right) - N_{B2} \cdot L_{eff} \left( {V_{D2} } \right)}}{{L_{eff} \left( {V_{D1} } \right) - L_{eff} \left( {V_{D2} } \right)}}$$

By repeating this procedure for various *V*_D_ values, the spatial distribution of the electrical defect concentration in the gate insulator can be progressively reconstructed along the channel direction. In conventional LFN extraction, the extracted *N*_B_ corresponds to a spatially averaged defect concentration over the effective channel region. Therefore, when represented along the channel direction, the conventional result is equivalent to assuming a uniform defect distribution over *L*_eff_, and localized source/drain-side non-uniformity cannot be resolved. In the present method, however, multiple averaged *N*_B_ values are obtained at different *L*_eff_ values by drain- or source-bias modulation. The difference between two successive averaged quantities is then used to calculate the defect concentration in the corresponding channel segment, as expressed in Eqs. (4) and (5). Thus, the proposed method reconstructs the lateral *N*_B_ distribution from a series of controlled *L*_eff_-dependent averages, rather than from a single channel-averaged value. For each pair of *V*_G_ and *V*_D_ (i.e., for a fixed *L*_eff_), *N*_B_ and the Coulomb scattering coefficient (*α*_C_) in the SA TG coplanar IGZO TFT are extracted using the LFN model established in our previous work^34–36^. In this approach, the measured drain current noise power spectral density (*S*_ID_) is fitted to Eq. (6) to determine *N*_B_ and *α*_C_.

6 $$\frac{{S_{{I_{D} }} }}{{I_{D}^{2} }} = \left( {1 + \alpha_{c} \mu_{FEi} C_{i} \frac{{I_{D} }}{{g_{mi} }}} \right)^{2} \left( {\frac{{R_{Ch} }}{{R_{T} }} \cdot \frac{{g_{m} }}{{I_{D} }}} \right)^{2} \frac{{q^{2} kT\lambda N_{B} }}{{f \cdot W \cdot L_{eff} \cdot C_{i}^{2} }}$$

where *g*_mi_ is the intrinsic transconductance, *R*_Ch_ is the channel resistance, *q* is the elementary charge, *k* is the Boltzmann constant, *T* is the temperature, *λ* is the tunneling attenuation length in the gate dielectric (= 10^−8^ cm)^37^, and *f* is the frequency. To minimize parameter correlation in the extraction, the parasitic resistance *R*_par_ and intrinsic field-effect mobility *μ*_FEi_ are first determined independently using the paired *V*_D_-based TLM from the linear-region current–voltage characteristics. Because these parameters are extracted solely from DC I–V data, they are not coupled to the noise model parameters *N*_B_ and *α*_C_. The independently obtained *R*_par_ and *μ*_FEi_ are then substituted into the modified CMF model expressed in Eq. (6), which incorporates the channel-specific characteristics of SA TG coplanar IGZO TFTs, leaving only *N*_B_ and *α*_C_ as free parameters. These two parameters are constrained by simultaneously fitting LFN spectra at three closely spaced gate-bias points. Within the model, *N*_B_ and *α*_C_ enter in distinct functional roles—*N*_B_ sets the overall noise magnitude, while *α*_C_ governs the bias dependence through Coulomb scattering— thereby reducing parameter correlation between *N*_B_ and *α*_C_.

In this work, *λ* was kept fixed for all source- and drain-side extractions to evaluate the lateral *N*_B_ distribution under a consistent LFN tunneling framework. Although local variations in trap properties may affect the absolute magnitude of the extracted *N*_B_, the same SiO_2_ gate dielectric and extraction framework were used for both source- and drain-side modulation, allowing the lateral trend to be compared under a common model assumption. To improve the extraction accuracy, measurements are performed at closely spaced gate voltages (*V*_G_ ± Δ*V*_G_), where Δ*V*_G_ is sufficiently small such that both *N*_B_ and *α*_C_ are assumed to remain unchanged. The LFN data obtained under these bias conditions are simultaneously fitted to Eq. (6), enabling reliable determination of *N*_B_ at each effective channel length. By defining *X*_B_ = √(1/*N*_B_), Eq. (6) can be rearranged into a linear form with *X*_B_ and *α*_C_ as the two unknowns (Eq. (S1)). The three LFN datasets measured at closely spaced *V*_G_ values then provide three linear relations, and their intersection gives a unique solution for *X*_B_ and *α*_C_. The defect concentration is subsequently obtained as *N*_B_ = 1/*X*_B_^2^.

## Results and discussion

The fabrication process began with the deposition of a 300-nm-thick SiO_2_ buffer layer on a glass substrate using plasma-enhanced chemical vapor deposition (PECVD). A 30-nm amorphous IGZO film (In:Ga:Zn = 1:1:1 mol%) was subsequently deposited at room temperature by DC magnetron sputtering and patterned through a wet chemical etching process to define the active region. A 150-nm SiO_2_ gate dielectric layer was then deposited via PECVD, followed by the formation of a Cu/MoTi gate electrode using DC sputtering. The gate metal layer was patterned by photolithography and subsequently removed by wet etching. Using the patterned gate photoresist as a mask, the underlying gate dielectric was selectively etched by a dry plasma process to form the gate stack structure. After gate stack definition, interlayer dielectric films composed of SiO_X_ and SiN_X_ were sequentially deposited by PECVD and patterned to open contact vias. Source and drain electrodes consisting of Cu/MoTi were then deposited and aligned with the gate electrode to realize a coplanar self-aligned configuration. Finally, a top passivation layer of SiO_2_ was deposited over the entire device structure, followed by thermal annealing to stabilize the electrical characteristics and improve film uniformity.

**Figure 3:**
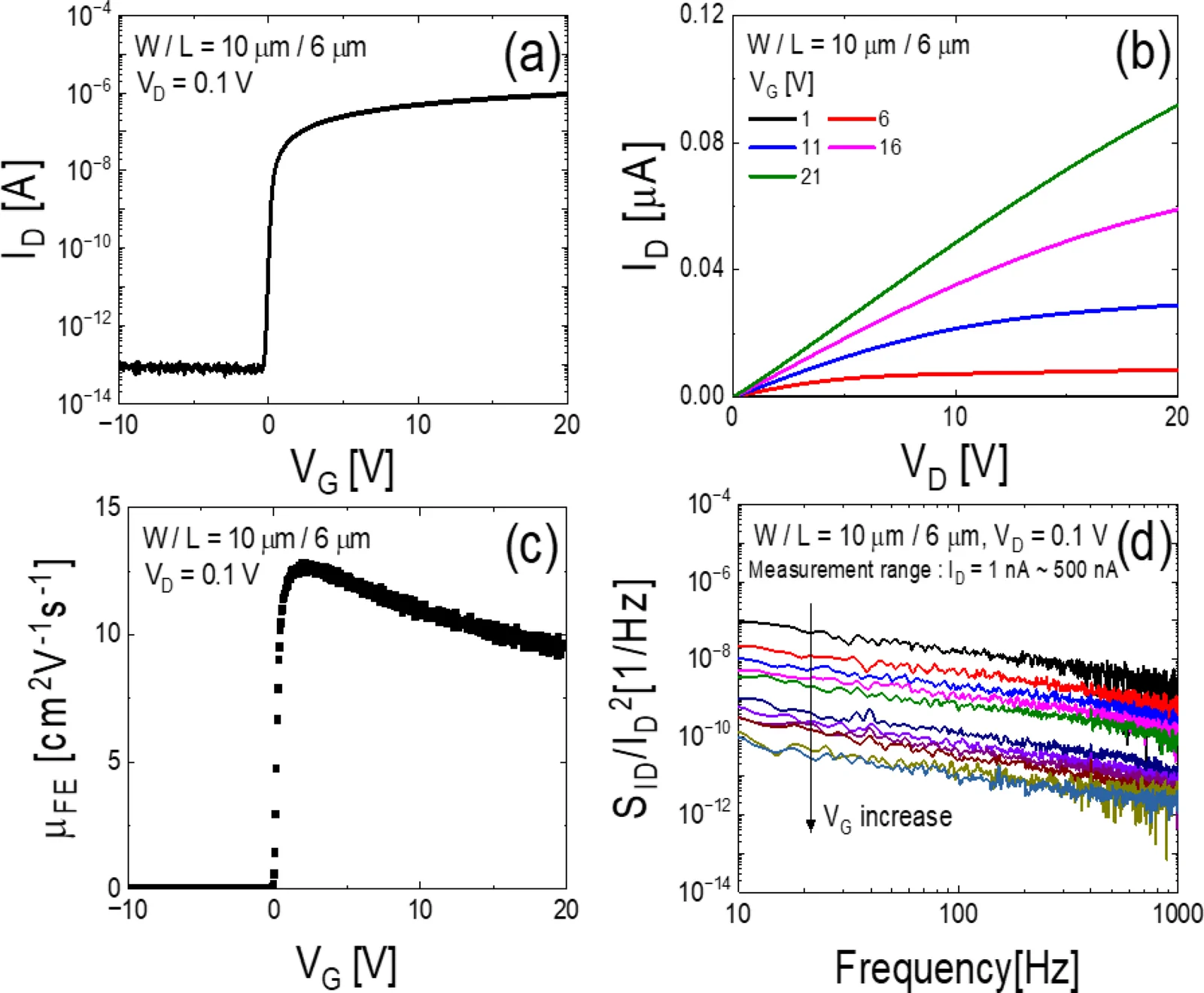
Electrical characteristics and LFN properties of the SA TG coplanar IGZO TFT (*W/L* = 10 μm/6 μm) obtained at RT. (**a**) Transfer characteristics at *V*_D_ = 0.1 V. (**b**) Output characteristics measured at various *V*_G_. (**c**) *μ*_FE_ extracted as a function of *V*_G_. (**d**) Frequency dependence of normalized *S*_ID_/*I*_D_^*2*^ measured at different *I*_D_ levels.

Figure 3 summarizes the electrical characteristics of the fabricated SA TG coplanar IGZO TFTs. Figure 3a,b present the transfer and output characteristics of a representative device with a channel width/length (*W/L*) of 10 μm/6 μm, measured at room temperature (RT). For the transfer characteristics, the gate voltage (*V*_G_) was swept from − 10 to 20 V while the drain voltage (*V*_D_) was fixed at 0.1 V, and the corresponding drain current (*I*_D_) was recorded. The output characteristics were obtained by sweeping *V*_D_ from 0 to 20 V at several fixed values of *V*_G_. All electrical measurements were performed using an Agilent 4156*C* semiconductor parameter analyzer. Figure 3c shows the field-effect mobility (*μ*_FE_ = *g*_m_·*L*/(*C*_i_·*V*_D_·*W*) extracted as a function of *V*_G_ at *V*_D_ = 0.1 V, where *g*_m_ denotes the transconductance. Figure 3d illustrates the frequency dependence of the normalized drain current noise power spectral density (*S*_ID_/*I*_D_^*2*^), measured at different *I*_D_ levels by sweeping *V*_G_ while maintaining *V*_D_ at 0.1 V for SA TG coplanar IGZO TFTs with *W/L* = 10 μm/6 μm. The LFN measurements were conducted under ambient conditions at RT using an Agilent 89,441 vector signal analyzer together with an SR570 low-noise current amplifier (Stanford Research Systems). Although amorphous IGZO can exhibit trap-limited and percolation-related conduction owing to its disordered subgap DOS^1,37^, the present LFN analysis was performed using a modified *C*MF model that experimentally extracted device parameters of the SA TG coplanar IGZO TFTs. Specifically, *R*_par_, *L*_eff_, and *μ*_FEi_ were extracted from the D*C* characteristics and incorporated into the noise model. Therefore, the proposed framework accounts for the source/drain parasitic resistance and effective-channel-length modulation inherent to this device architecture, enabling *N*_B_ and *α*_C_ to be extracted using a device-specific LFN model.

**Figure 4:**
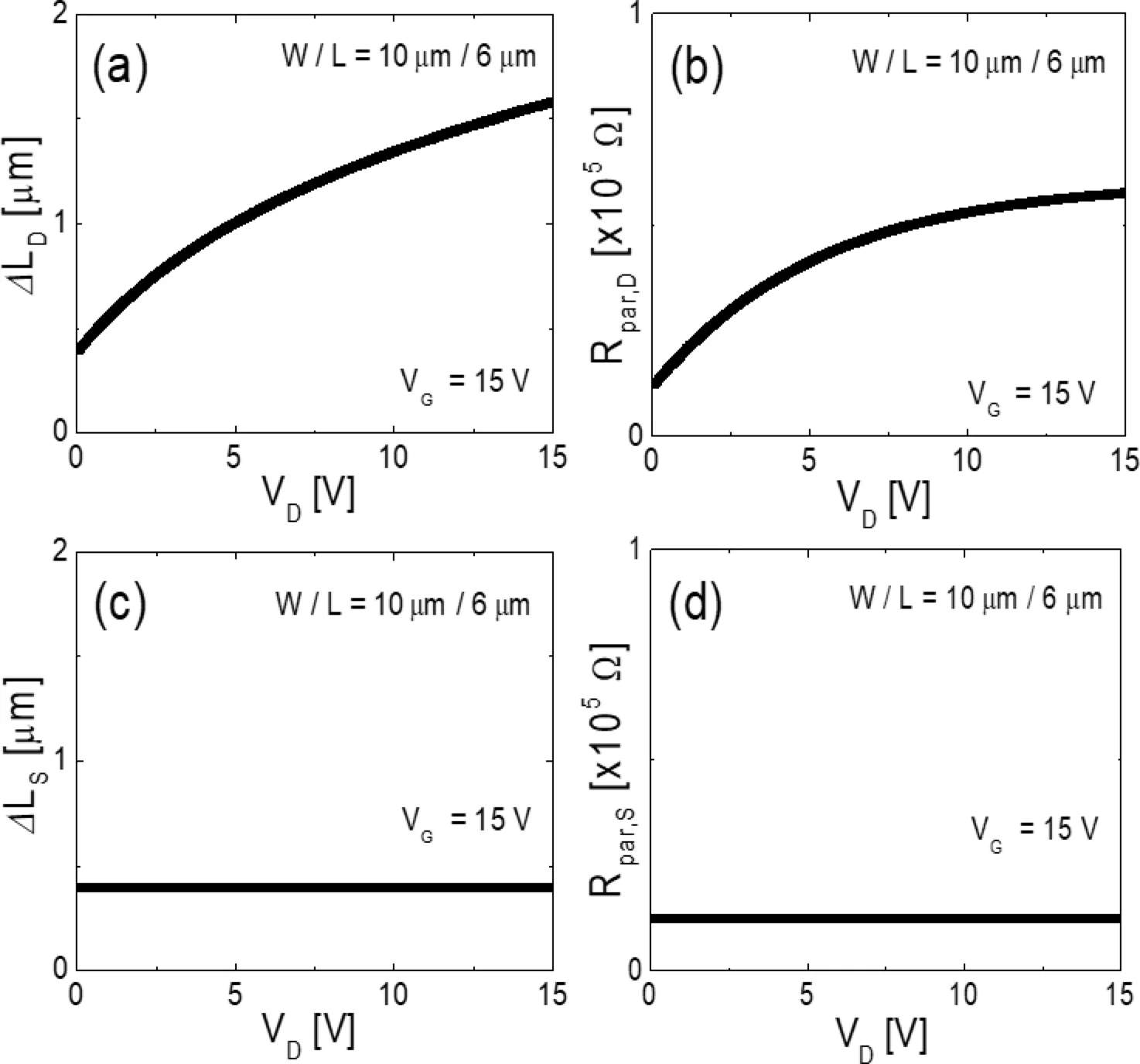
Extraction of *ΔL* and *R*_par_ using the paired *V*_D_-based TLM at *V*_G_ = 15 V. (**a**) *ΔL*_D_ and (**b**) *R*_par,D_ as functions of *V*_D_. (**c**) *ΔL*_S_ and (**d**) *R*_par,S_ under varying *V*_D_, indicating that these parameters are independent of the drain bias.

Figure 4 illustrates the extraction results of the source/drain-side channel shortening (*ΔL*_S_, *ΔL*_D_) and parasitic resistance (*R*_par,S_, *R*_par,D_) as functions of *V*_D_ at a fixed *V*_G_ = 15 V. As discussed in the Methods section, these parameters were determined using the paired *V*_D_-based TLM. To implement this extraction, a set of devices with varying drawn gate lengths (*L* = 4, 4.5, and 6 μm) was utilized, while ensuring that the device operation remained within the linear regime for the validity of the extraction model. As shown in Fig. 4a, *ΔL*_D_ exhibits a clear dependence on the applied drain bias, increasing significantly as *V*_D_ rises. This trend is consistent with the physical mechanism depicted in Fig. 1, where an increase in *V*_D_ reduces *V*_GD_, thereby lowering the induced electron concentration and causing the effective channel boundary to retreat toward the source. Consequently, the drain-side parasitic resistance *R*_par,D_ also shows a corresponding increase with *V*_D_, as shown in Fig. 4b. In contrast, the source-side parameters *ΔL*_S_ and *R*_par,S_ remain nearly constant regardless of the *V*_D_ variations, as presented in Fig. 4c,d because *V*_GS_ remains fixed at 15 V (with *V*_S_ = 0 V), making the electron concentration profile near the source electrode independent of the applied *V*_D_.

**Figure 5:**
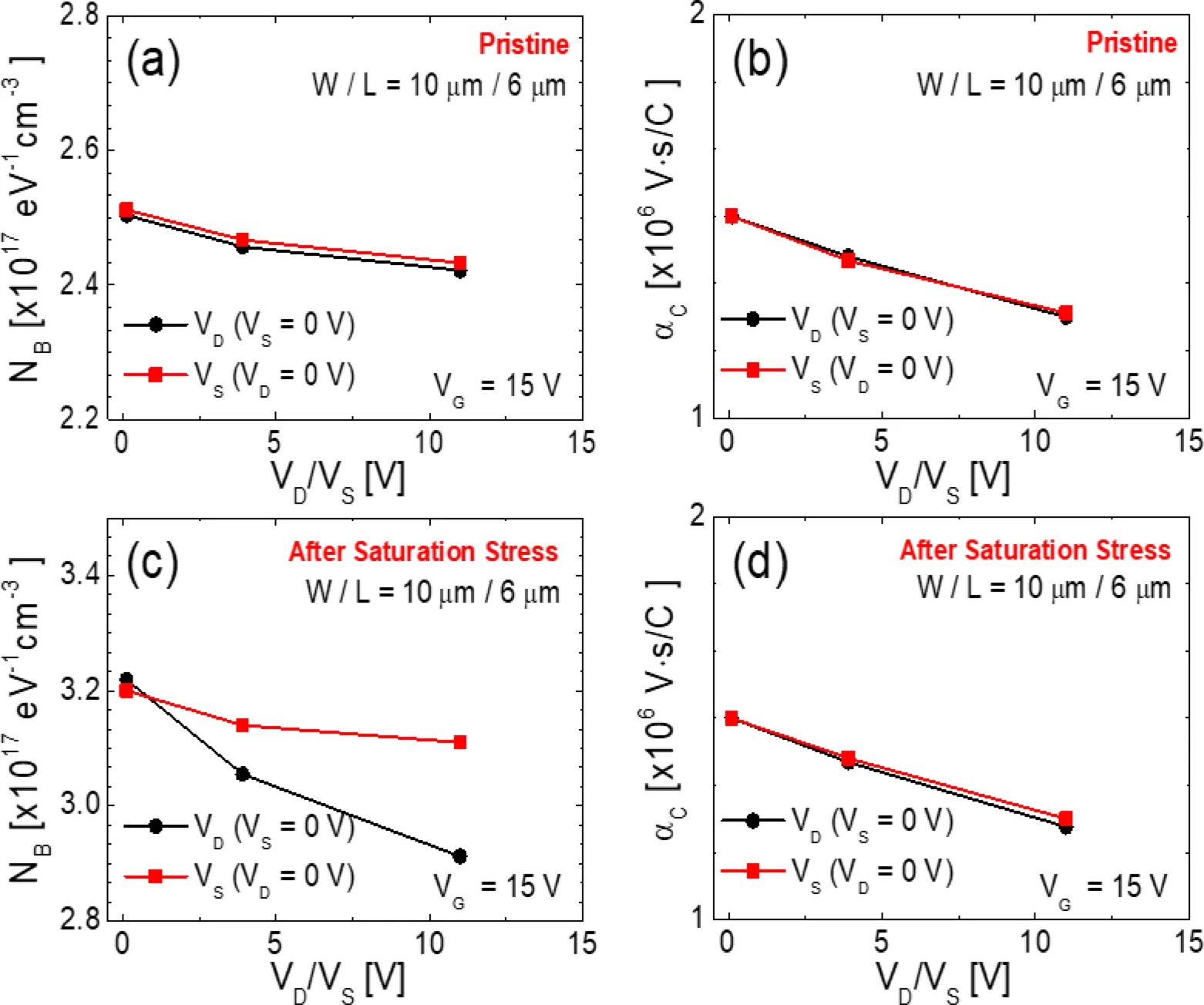
Extracted *N*_B_ and *α*_C_ for pristine and stressed devices. (**a**, **b**) *N*_B_ and *α*_C_ for the pristine device under drain- and source-bias modulation. (**c**, **d**) *N*_B_ and *α*_C_ after saturation stress (*V*_G_ = 5 V, *V*_D_ = 20 V, 1500 s). The *N*_B_ dependence on *V*_D_ after stress reflects the lateral non-uniformity of the defect distribution.

Figure 5 presents the extracted values of *N*_B_ and *α*_C_ under drain- and source-bias modulation for both the pristine device and the device after saturation stress (*V*_G_ = 5 V, *V*_S_ = 0 V, *V*_D_ = 20 V, stress time 1500 s, at RT). Before analyzing the post-stress LFN characteristics, we examined whether the saturation stress significantly changed the basic transport characteristics or the parasitic resistance. As shown in Fig. S1(a), the transfer characteristics and field-effect mobility show negligible changes after saturation stress (*V*_G_ = 5 V, *V*_D_ = 20 V, 1500 s). Because a substantial degradation of the n^+^ extension regions would increase the series resistance and reduce the apparent linear-regime current or mobility, this result suggests that stress-induced access-region degradation is limited. In addition, the *R*_par_ values extracted by MELM are in good agreement with those obtained by the *V*_G_-based TLM, validating the use of MELM for single-device monitoring [Fig. S1(b)]. The MELM-extracted *R*_par_ before and after saturation stress also shows negligible change [Fig. S1(c)]. Therefore, the stress-induced increase in *N*_B_ near the drain side is unlikely to originate from parasitic-resistance degradation. Figure 5a,b show the extracted *N*_B_ and *α*_C_, respectively, for the pristine device, while Fig. 5c,d present the corresponding results after the application of saturation stress. In these measurements, *V*_G_ was fixed at 15 V while either *V*_D_ or *V*_S_ was varied to modulate the effective channel boundary on the drain or source side. For the drain-bias modulation (*V*_S_ = 0 V), the measurements were performed at *V*_D_ = 0.1 V, 3.9 V, and 11 V. Conversely, for the source-bias modulation (*V*_D_ = 0 V), the measurements were performed at *V*_S_ = 0.1 V, 3.9 V, and 11 V, providing symmetric bias conditions for probing the source-side effective channel modulation. For each specified bias condition (i.e., fixed *V*_G_ and *V*_D_ (or *V*_S_) corresponding to a particular *L*_eff_), *N*_B_ was extracted using the *V*_G_ ± Δ*V*_G_-based LFN analysis method described in the Methods section. Specifically, LFN spectra were measured at closely spaced gate voltages (*V*_G_ = 13, 15, 17 V), which were selected such that device operation remained within the linear regime for all applied drain or source bias conditions. The normalized noise characteristics were simultaneously fitted under the assumption that *N*_B_ and *α*_C_ remain unchanged within this small gate-bias interval. Using this procedure, *N*_B_ and *α*_C_ were extracted for each effective channel length for both the pristine and stressed devices. As shown in Fig. 5c,d, the extracted parameters exhibit a clear dependence on the applied lateral bias, reflecting the lateral non-uniformity of the gate-insulator defect distribution along the channel direction. In particular, after the application of saturation stress, the extracted *N*_B_ decreases as *V*_D_ increases while *V*_S_ is fixed at 0 V. This behavior indicates that the degradation of the gate insulator is spatially localized near the drain-side region of the channel. As *V*_D_ increases, the drain-side effective channel boundary moves toward the source, resulting in a progressive reduction of the effective channel length. Consequently, the degraded gate-insulator region near the drain becomes increasingly excluded from the effective channel region used for the extraction of *N*_B_, leading to a decrease in the extracted average defect concentration.

**Figure 6:**
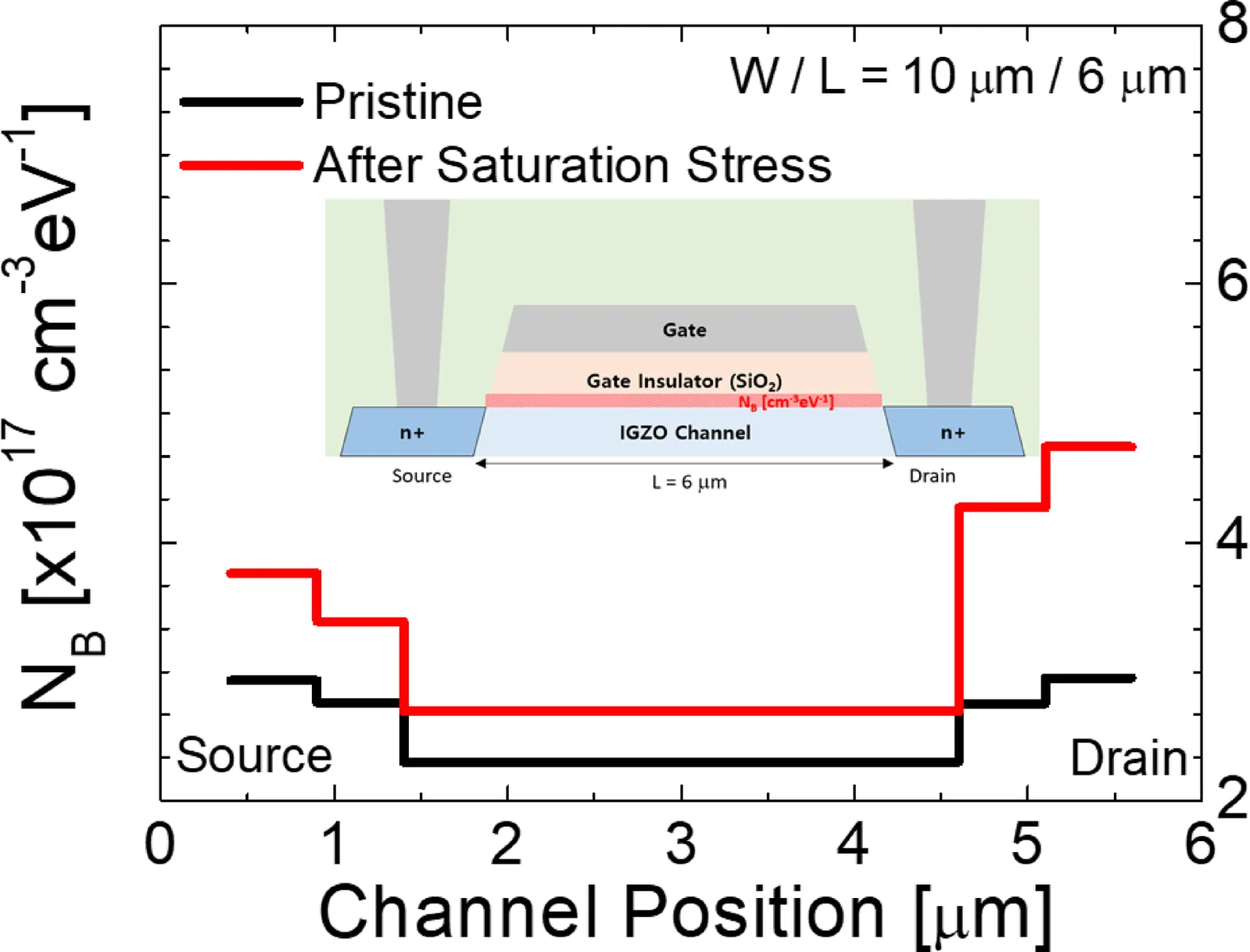
Extracted lateral distribution of *N*_B_ along the channel direction for the SA TG coplanar IGZO TFT (*W/L* = 10 μm/6 μm). The black and red curves represent the pristine device and the device after saturation stress, respectively. The pronounced increase in *N*_B_ near the drain region after stress indicates localized degradation of the gate insulator.

Figure 6 shows the extracted lateral distribution of *N*_B_ along the channel direction for the SA TG coplanar IGZO TFT with *W/L* = 10 μm / 6 μm. The distributions were obtained by substituting the extracted parameters shown in Figs. 3, 4 and 5 into equations described in the Methods section. The black and red curves represent the pristine device and the device after saturation stress, respectively. For the pristine device, the extracted *N*_B_ shows a relatively uniform distribution along the channel, with slightly higher values near the source and drain sides. This behavior is consistent with the expected plasma exposure of the gate insulator outside the gate electrode during the self-aligned gate-insulator etching process. After the application of saturation stress, a pronounced increase in the defect concentration is observed near the drain side of the channel. This result indicates that the degradation of the gate insulator is localized near the drain region, where a large lateral electric field is present during saturation operation. A less pronounced increase in *N*_B_ is also observed near the source side, which may be attributed to the relatively large vertical gate bias experienced by the channel region near the source during the saturation stress condition. These results demonstrate that the proposed LFN-based method enables the experimental extraction of the lateral distribution of gate insulator defects in SA TG coplanar IGZO TFTs. The ability to resolve the spatial localization of defect generation provides useful insight into reliability degradation mechanisms in oxide TFT technologies. It should be noted that the present LFN measurement was performed within a finite frequency range; therefore, the extracted *N*_B_ represents gate-insulator defects that contribute to the measured LFN within the adopted tunneling framework. Since the characteristic time constant of oxide traps depends on their tunneling distance from the channel/dielectric interface, a broader LFN frequency range would provide more detailed time-constant-resolved information and allow a more refined apparent probing-depth analysis. Accordingly, the present *N*_B_ profile should be interpreted as the lateral distribution of LFN-active gate-insulator defects within the measured frequency window.

## Conclusion

In this work, a method for experimentally extracting the lateral distribution of electrical defect concentration in the gate insulator of SA TG coplanar IGZO TFTs was developed based on LFN analysis. By modulating the effective channel length through drain- and source-bias control and combining the extracted average defect concentrations corresponding to different effective channel lengths, the spatial distribution of gate insulator defects along the channel direction was obtained. The extracted defect profiles indicate that the pristine device exhibits a relatively uniform distribution across the channel, with slightly elevated values near the source and drain regions, which is consistent with the expected plasma exposure during the self-aligned gate-insulator etching process. After saturation stress, a pronounced increase in defect concentration is observed near the drain region, suggesting that the degradation of the gate insulator is strongly localized in the high lateral electric-field region. The proposed LFN-based analysis provides a practical experimental approach for resolving the lateral distribution of gate-insulator defects contributing to the measured LFN in SA TG coplanar IGZO TFTs, which has been difficult to achieve using conventional techniques developed for silicon MOSFETs. This approach offers useful insight into degradation mechanisms and may serve as an effective diagnostic tool for improving device reliability and process design in advanced oxide TFT technologies.

## Supplementary Information

Below is the link to the electronic supplementary material.


Supplementary Material 1.


## Data Availability

The datasets used and/or analyzed during the current study are available from the corresponding author on reasonable request.
