# Supplementary material for: Lateral distribution of electrical defect concentration in SA TG coplanar IGZO TFTs extracted via low frequency noise analysis
Source: Sci Rep. 2026 Jun 21;16:27259. doi: 10.1038/s41598-026-58574-z (PMC13530169; doi:10.1038/s41598-026-58574-z)
Supplement: Supplementary file 1 [file 41598_2026_58574_MOESM1_ESM.docx]

**Supplementary Information**

**Lateral Distribution of Electrical Defect Concentration in SA TG Coplanar IGZO TFTs Extracted via Low Frequency Noise Analysis**

Dong-Ho Lee^1,+^, Jin-Ha Hwang^1,+^, Chae-Eun Oh^1^, Jihee Ryu^2^, Jae-Man Jang^2^, Byung-Du Ahn^2^, Jong-Uk Bae^2^, and Hyuck-In Kwon^1,*^

^1^Major in Intelligent Semiconductor Engineering, Chung-Ang University, 84 Heukseok-ro, Dongjak-gu, Seoul, Korea

^2^Research and Development Center, LG Display Company, Paju 10845, Korea

^*^corresponding author: Hyuck-In Kwon (hyuckin@cau.ac.kr)

^+^these authors contributed equally to this work

**Figure S1. Verification of transport and parasitic-resistance stability after saturation stress.**

**(a) Transfer characteristics before and after saturation stress** (*V*_G_ = 5 **V,** *V*_D_ = 20 **V, 1500 s); the inset shows the corresponding field-effect mobility. (b) Comparison of** *R*_par_ extracted **by the** *V*_G_-based **TLM and MELM. (c) MELM-extracted** *R*_par_ before **and after saturation stress.**

**Equation S1. Linearized form for extracting *N*_B_ and *α*_C_.**
For the simultaneous extraction of *N*_B_ and *α*_C_, Eq. (6) in the main text can be rearranged into a linear form by defining

$$X_{B}=\sqrt{\frac{1}{N_{B}}}, Y=\alpha_{C}$$

The measured LFN data at each *V*_G_ then provide one linear relation between *X*_B_ and *Y*:

$$M=\frac{S_{I_{D}}}{I_{D}^{2}},A=\mu_{eff}C_{ox}\frac{I_{D}}{g_{mi}},B=\frac{R_{Ch}}{R_{tot}}\frac{g_{m}}{I_{D}},C=\frac{q^{2}kT\lambda}{fWL_{eff}C_{ox}^{2}}$$

$$M=B^{2}CN_{B}(1+AY)^{2}, \sqrt{M}X_{B}=B\sqrt{C}\left( 1+AY \right)$$

$$Y=\frac{\sqrt{M}}{AB\sqrt{C}}X_{B}-\frac{1}{A}=\frac{\sqrt{\frac{S_{I_{D}}}{I_{D}^{2}}}}{\mu_{eff}\frac{g_{m}}{g_{mi}}\frac{R_{Ch}}{R_{tot}}q\sqrt{\frac{kT\lambda}{fWL_{eff}}}}X_{B}-\frac{g_{mi}}{\mu_{eff}C_{ox}I_{D}} (S1)$$

Because the LFN spectra were measured at three closely spaced gate voltages (*V*_G_−Δ*V*_G_, *V*_G_, *V*_G_+Δ*V*_G_), three linear relations are obtained. The intersection of these lines gives a unique pair of *X*_B_ and *α*_C_, from which *N*_B_=1/*X*_B_*^2^* is calculated.
